# Novel Gallate Triphenylphosphonium Derivatives with Potent Antichagasic Activity

**DOI:** 10.1371/journal.pone.0136852

**Published:** 2015-08-28

**Authors:** Leonel A. Cortes, Lorena Castro, Bárbara Pesce, Juan D. Maya, Jorge Ferreira, Vicente Castro-Castillo, Eduardo Parra, José A. Jara, Rodrigo López-Muñoz

**Affiliations:** 1 Programa de Farmacología Molecular y Clínica, ICBM, Facultad de Medicina, Universidad de Chile, Santiago, Chile; 2 Departamento de Química, Facultad de Ciencias Básicas, Universidad Metropolitana de Ciencias de la Educación, Santiago, Chile; 3 Laboratory of Experimental Biomedicine, University of Tarapacá, Iquique, Chile; 4 Unidad de Farmacología y Farmacogenética, ICOD, Facultad de Odontología, Universidad de Chile, Santiago, Chile; 5 Instituto de Farmacología y Morfofisiología, Facultad de Ciencias Veterinarias, Universidad Austral de Chile, Valdivia, Chile; University of Melbourne, AUSTRALIA

## Abstract

Chagas disease is one of the most neglected tropical diseases in the world, affecting nearly 15 million people, primarily in Latin America. Only two drugs are used for the treatment of this disease, nifurtimox and benznidazole. These drugs have limited efficacy and frequently induce adverse effects, limiting their usefulness. Consequently, new drugs must be found. In this study, we demonstrated the *in vitro* trypanocidal effects of a series of four gallic acid derivatives characterized by a gallate group linked to a triphenylphosphonium (TPP^+^) moiety (a delocalized cation) *via* a hydrocarbon chain of 8, 10, 11, or 12 atoms (TPP^+^-C_8_, TPP^+^-C_10_, TPP^+^-C_11_, and TPP^+^-C_12_, respectively). We analyzed parasite viability in isolated parasites (by MTT reduction and flow cytometry) and infected mammalian cells using *T*. *cruzi* Y strain trypomastigotes. Among the four derivatives, TPP^+^-C_10_ and TPP^+^-C_12_ were the most potent in both models, with EC_50_ values (in isolated parasites) of 1.0 ± 0.6 and 1.0 ± 0.7 μM, respectively, and were significantly more potent than nifurtimox (EC_50_ = 4.1 ± 0.6 μM). At 1 μM, TPP^+^-C_10_ and TPP^+^-C_12_ induced markers of cell death, such as phosphatidylserine exposure and propidium iodide permeabilization. In addition, at 1 μM, TPP^+^-C_10_ and TPP^+^-C_12_ significantly decreased the number of intracellular amastigotes (TPP^+^-C_10_: 24.3%, TPP^+^-C_12_: 19.0% of control measurements, as measured by DAPI staining) and the parasite’s DNA load (C_10_: 10%, C_12_: 13% of control measurements, as measured by qPCR). Based on the previous mode of action described for these compounds in cancer cells, we explored their mitochondrial effects in isolated trypomastigotes. TPP^+^-C_10_ and TPP^+^-C_12_ were the most potent compounds, significantly altering mitochondrial membrane potential at 1 μM (measured by JC-1 fluorescence) and inducing mitochondrial transition pore opening at 5 μM. Taken together, these results indicate that the TPP^+^-C_10_ and TPP^+^-C_12_ derivatives of gallic acid are promising trypanocidal agents with mitochondrial activity.

## Introduction

Chagas disease (American trypanosomiasis) is a parasitic illness caused by *Trypanosoma cruzi*, a flagellate protozoan [1]. Chagas disease has been present in the Americas for 9,000 years, and its range extends from the southern United States to the central regions of Chile and Argentina [2]. Chagas disease is a serious issue in Latin America, where it is one of the parasitic diseases with the highest prevalence and mortality. Overall, the economic burden caused by this disease in Latin America is devastating, as it is responsible for the loss of 670,000 years of disability-adjusted life [3]. Despite public health initiatives and vector control strategies, the infected population in the Americas is estimated to be 8 million individuals, with 50,000 new cases recorded each year [4]. In addition, due to the migration of individuals from endemic regions, which increases the number of people at risk for infection, there is now concern regarding the “global problem” of Chagas disease. Under these circumstances, the infection is primarily spread by blood and organ transplants in countries such as the USA, Japan, and Australia, where blood banks do not have control mechanisms for detecting the parasite [5, 6].

There is no vaccine for preventing the infection, and the only drugs that are currently used for the treatment of Chagas disease are nifurtimox (Lampit, Bayer) and benznidazole (Rochagan, Roche). These drugs were empirically developed in the early 1960s, and both drugs have limited efficacy and induce several adverse effects, ranging from abdominal discomfort to leukopenia or peripheral neuropathy [7]. These limitations have stimulated research on novel therapeutic strategies to improve the treatment of this disease.

Over the past decade, mitochondria have become interesting pharmacological targets for the treatment of many pathologies, such as cancer and neurodegenerative diseases [8]. Indeed, the metabolic role of mitochondria makes them a pivotal organelle in eukaryotic cells, and this role can be explained by their wide distribution, large number, and diverse functions. In contrast to mammalian cells, *Trypanosoma cruzi* has a single mitochondrion that exhibits some particular features, such as a region rich in DNA termed the kinetoplast [9], an internal rotenone-insensitive NADH dehydrogenase, the absence of NADH dehydrogenase coupled to phosphorylation site I, and a branched electron transport chain that permits cyanide-resistant respiration, which confers significant flexibility to the respiratory chain. This respiration could also be mediated by an alternative oxidase, similar to the alternative oxidase found in *T*. *brucei* [10], which has been observed biochemically in *T*. *cruzi* [11]; its gene and mRNA have also been identified (GenBank accession number: AB189129.1). Mitochondria maintain energy production and the key systems of metabolite synthesis. In aerobic environments, eukaryotic mitochondria obtain their energy primarily through oxidative phosphorylation *via* the electron transport chain (ETC), which consists of four enzyme complexes within the inner mitochondrial membrane. Complexes I and II act as electron acceptors (NADH + H^+^ and FADH_2_); complexes I, III, and IV act as H^+^ pumps, generating a proton electrochemical gradient that drives ATP synthesis *via* ATP synthase activity (Complex V). However, recent finding demonstrate that complex I of the respiratory chain has limited functions in *T*. *cruzi* metabolism [12, 13]. Moreover, succinate has been shown to be the main substrate that supports oxygen consumption in epimastigotes [14, 15].

In addition, electron transport and these electrochemical gradients contribute to the maintenance of mitochondrial transmembrane potential (ΔΨm) and the regulation of intracellular Ca^+2^ concentrations. Thus, ΔΨm maintenance represents the functional status of the mitochondrion both in eukaryotic cells and trypanosomatids [16, 17]. Alterations in mitochondrial membrane potential are a consequence of diverse cellular events, such as ETC inhibition, ATP synthase activity blockade, uncoupling protein stimulation, or inner membrane permeabilization by uncoupling agents [18]. The relevance of parasite mitochondria as a trypanocidal target is evidenced by the biological activity of several mitochondrial inhibitors, which have been shown to cause functional alterations in mitochondria, a loss in ATP production, and apoptosis in these parasites [19–22].

Due to the importance of mitochondria in several diseases, the triphenylphosphonium (TPP^+^) moiety has been used as a precursor to synthesize lipophilic cations with the aim of targeting small molecules to mitochondria [23, 24]. One of the most common uses of TPP^+^ derivatives is to improve the mitochondrial tropism of antioxidants agents [25, 26]. Gallic acid (GA) is a polyhydroxy phenol with known antioxidant and ETC inhibition properties. In addition, GA and its esters are able to inhibit cellular respiration in *T*. *cruzi*, inducing changes in motility and shape and resulting in lysis of the parasite [27].

The negative charge of the inner face of the parasite’s inner mitochondrial membrane (IMM) could attract lipophilic cations with TPP^+^ moieties [28]. Therefore, GA linked with a TPP^+^ moiety *via* a hydrocarbon chain could be used to transport trypanocidal compounds such that they would selectively accumulate within *T*. *cruzi* mitochondria and exert cytotoxic effects. In this case, the alkyl chain of the final molecule is more lipophilic than the TPP+ moiety alone, which compensates for the relatively hydrophilic nature of the trihydroxybenzoic acid group. This makes the group a delocalized lipophilic cation with the main characteristic that despite being a charged molecule (delocalized charge), its high lipophilicity allows it to cross barriers such as plasma and outer mitochondrial membranes [24, 29]. The design of such molecules with different chain lengths meets the need to evaluate a suitable chain length to allow better intercalation of the cationic compounds in the inner mitochondrial membrane or the possibility of crossing the IMM and reaching the mitochondrial matrix. Because of their positive charge, cations in the cytoplasm are attracted to the mitochondrial membrane and accumulate selectively within the IMM, exhibiting a 500-fold increase in concentration at this membrane. [30]. Accordingly, this process could alter vital mitochondrial function.

Recently, we synthesized four TPP^+^ GA derivatives, linking both moieties with hydrocarbon chains of 8, 10, 11, and 12 carbon atoms. These derivatives showed selective antitumor activity *in vitro*, with a mode of action involving altered mitochondrial function [31]. In the present study, we explored the trypanocidal effect of these newly synthesized TPP^+^ GA derivatives in two *in vitro* models: isolated parasites and infected mammalian cells. In addition, we explored alterations in the trypomastigote mitochondrial membrane as an indicator of mitochondrial activity.

## Materials and Methods

### 2.1. Drugs

**Fig 1:**
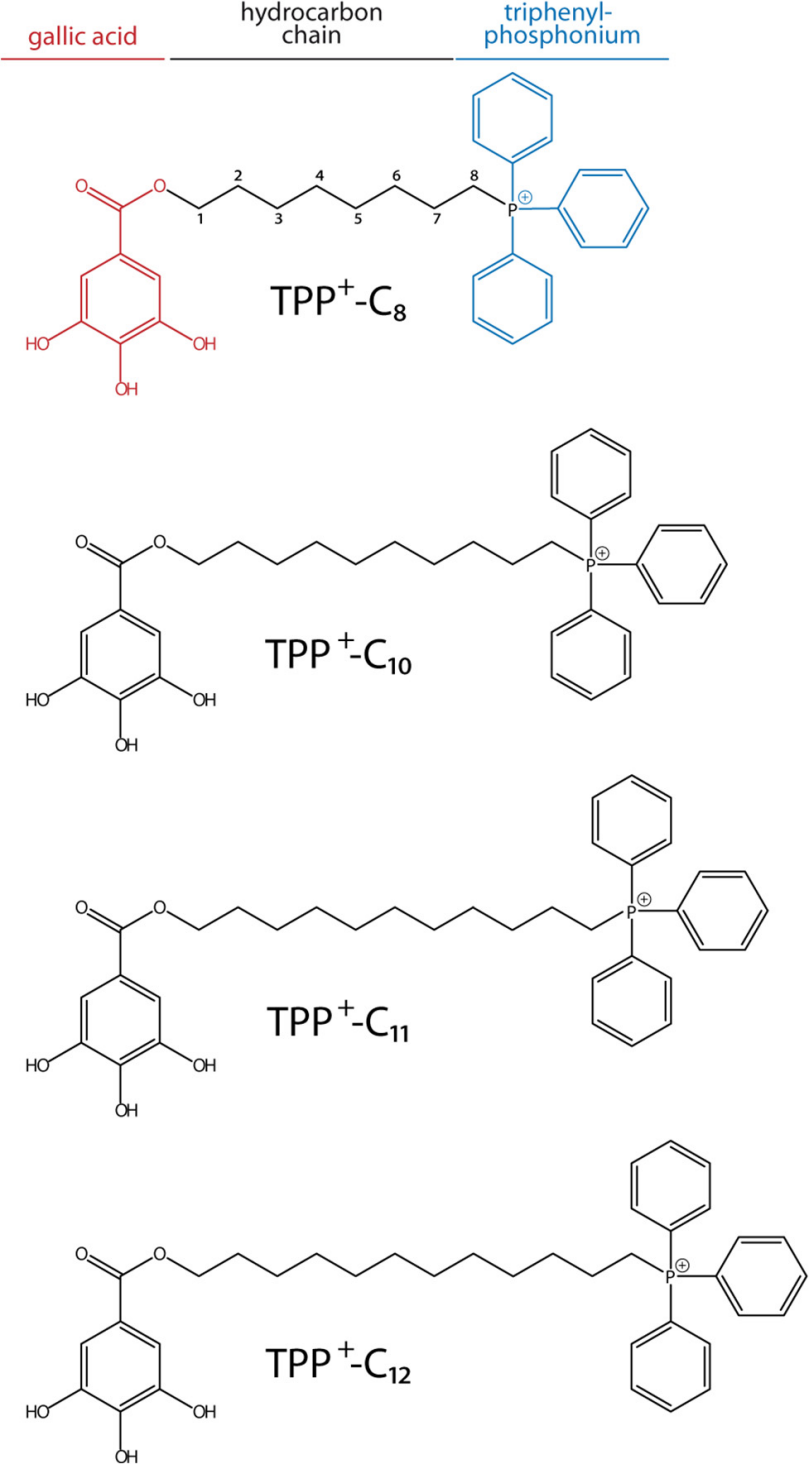
Chemical structure of the TPP+ derivatives. The structures of triphenyl(8-((3,4,5-trihydroxybenzoyl)oxy)-octyl) phosphonium bromide (TPP^+^-C_8_), triphenyl(10-((3,4,5-trihydroxybenzoyl)oxy)-decyl) phosphonium bromide (TPP^+^-C_10_), triphenyl(11-((3,4,5-trihydroxybenzoyl)oxy)-undecyl) phosphonium bromide (TPP^+^-C_11_), and triphenyl(12-((3,4,5-trihydroxybenzoyl)oxy)-dodecyl) phosphonium bromide (TPP^+^-C_12_) are shown. For details regarding their synthesis, see Jara *et al* (2014).

Nifurtimox was purchased from Sigma-Aldrich (Saint Louis, MO, USA). The gallate triphenylphosphonium derivatives (Fig 1), triphenyl(8-((3,4,5-trihydroxybenzoyl)oxy)-octyl) phosphonium bromide (TPP^+^-C_8_), triphenyl(10-((3,4,5-trihydroxybenzoyl)oxy)-decyl) phosphonium bromide (TPP^+^-C_10_), triphenyl(11-((3,4,5-trihydroxybenzoyl)oxy)-undecyl) phosphonium bromide (TPP^+^-C_11_), and triphenyl(12-((3,4,5-trihydroxybenzoyl)oxy)-dodecyl) phosphonium bromide (TPP^+^-C_12_), were synthesized in our laboratory, as previously described [31].

### 2.2. Parasites

Trypomastigotes (Y strain, DTU *T*. *cruzi* II) were obtained from infected VERO cells (*Chlorocebus sabaeus* kidney fibroblasts obtained from ATCC, ATCC number: CCL-81). The cells were exposed to trypomastigotes (Y strain) at a 3:1 density (trypomastigote:cell). The trypomastigotes were allowed to infect the cells for 24 h, and the supernatant was then extracted. The trypomastigotes were released from VERO cells at 4 days after infection. The parasites were harvested and collected for viability assays.

### 2.3. Viability Measurement Using the Tetrazolium Reduction Assay

The effect of the drugs on parasite viability was evaluated using the tetrazolium salt (MTT) reduction assay [32]. Parasites (10^7^/mL, cultured in a 24-well plate) or VERO cells (5 x 10^5^/mL, cultures in a 96-well plate) were incubated with the different compounds in RPMI-1640 medium, without phenol red, for 24 hours. After that, 100 μL of the parasite suspension was placed in a 96-well plate and incubated with 10 μL of a mixture containing 5 mg/mL MTT dye (3[4,5-dimethylthiazol-2-yl]-2,5-diphenyltetrazolium bromide) (Sigma USA) and 0.22 mg/mL phenazine methosulfate (Sigma USA) as an electron carrier. Alternatively, VERO cells attached to the surface of 96-well plates were washed and incubated with MTT (5 mg/mL). After incubation for 4 h at 37°C, the generated formazan crystals were dissolved with 100 μL of 10% (w/v) sodium dodecyl sulfate (SDS) in 0.01 M HCl. The plates were then incubated overnight at 37°C, and the optical density (OD) was determined using a microplate reader (Labsystems Multiskan MS, Finland) at 570 nm. Under these conditions, the OD is directly proportional to the number of viable cells in each well [33, 34]. All of the experiments were performed at least three times. Each experiment was performed in triplicate, and the drugs were assayed using at least five concentrations per experiment, ranging from 0.01 to 100 μM. The data from each experiment were plotted using the formazan absorbance versus the log of the concentration, and the plot was fitted to the dose-response (four-parameters) equation using the software Graph Pad Prism V5.0, obtaining the EC_50_ value per drug and per experiment. The data are expressed as the mean ± DS of the EC_50_s obtained from each experiment.

### 2.4. Flow Cytometry Cell Death Assays

For these assays, 10^7^ trypomastigotes (Y strain) were exposed to pentamidine for 24 h in RPMI at 37°C and 5% CO_2_. The cells were then washed in phosphate-buffered saline (PBS), resuspended, and incubated with propidium iodide (PI) and Annexin-V labeled with Alexa Fluor 488. The incubation was performed using Alexa Fluor 488 Annexin V/Dead Cell Apoptosis Kit (Molecular Probes, USA) following the manufacturer’s instructions. The fluorescence of the samples was measured using a FACSAria-III flow cytometer (BD biosciences, USA) with 530/30 and 630/22 filters to detect Annexin-V and PI, respectively. For each sample, 5,000 events were recorded, and the data were analyzed using FACSDiva 6.1 software.

### 2.5. DAPI Staining and Intracellular Amastigote Quantification

VERO (*Chlorocebus sabaeus* kidney fibroblasts, ATCC number: CCL-81) cells were exposed to *T*. *cruzi* trypomastigotes (Y strain) at a 5:1 ratio (trypomastigotes:cells) for 24 hours. The cells were then washed and treated with different compounds, at 0.1 and 1 μM, for 48 hours. The cells were washed and fixed in cold methanol (70%) overnight; the fixed cells were washed, and 1 mL of PBS (pH 7.4) was added. The DNA was stained with DAPI (NucBlue, Molecular Probes, USA) following the manufacturer’s instructions. The cells were photographed using a Nikon Eclipse 400 fluorescence microscope at 358 nm (excitation) and 461 nm (emission). Five images were obtained per well, and each picture was analyzed by two independent researchers.

### 2.6. Real-Time PCR

DNA from infected RAW cells (murine macrophages, ATCC number: TIB-71) was isolated using Wizard Genomic DNA Purification Kit (Promega, USA) following the manufacturer’s instructions. DNA was quantified through 280-nm absorbance measurements using a Varioskan spectrophotometer (Thermo Scientific, USA). To evaluate the parasite DNA load, we used a TaqMan-Duplex system, as described previously [35]. To amplify an 84-bp *T*. *cruzi* satellite DNA sequence, we used the following primers: TcSt 4-Fw (5’-GGACCACAACGTGTGATGCA-3’) and TcSt 1-Rev (5’-AGGAATTTCGCGAGCTCTTG-3’) and the TcSt-1 probe (5’-FAM-ATCAGCCGAGTGCAGCACCCTTG-BHQ-1-3’). As an endogenous control, we used the Mus-F (5’-GCAAAGCCTGACAACTTCTGAA-3’) and Mus-R (5’-CCAACGTCCCAGCTTAAGTAGAAT-3’) primers coupled with the MM-1 probe (5’-HEX-AAAGCATCTGCCTCCG-BHQ-1-3’) to amplify a 67-bp *Mus musculus* GAPDH sequence. The primers and probes were designed using Primer Express 3.0 Software (Applied Biosystems, USA) and manufactured by Integrated DNA Technologies (Coralville, IA, USA). The PCR reactions were performed using an ABI7300 real-time thermocycler (Applied Biosystems, USA). The reaction mixture had a final volume of 20 μL and contained 10 ng of genomic DNA, 4 μL of HOT FIREPol Probe qPCR Mix Plus (Solis BioDyne, Tallinn, Estonia), 200 nM of each primer, and 100 nM of the TcSt-1 probe or 200 nM of the MM-1 probe. ROX was used as a reference dye. For both TaqMan assays, the thermal cycle consisted of a polymerase activation step carried out at 95°C for 10 min (one cycle) and a two-step amplification phase: 95°C for 15 s and 55°C for 45 s (40 cycles). Fluorescence was measured at the end of each amplification cycle. The data were analyzed using 7300 System SDS software with the SDS relative quantitation plug-in (Applied Biosystems, USA). All of the data were analyzed by the 2^-∆∆CT^ method [36], comparing the C_T_s of the *T*. *cruzi* gene versus that of GAPDH as well as comparing all of the samples with the controls. The parasite load is expressed as the relative DNA load compared with the controls.

### 2.7. Mitochondrial Membrane Potential Assessment by JC-1

For the determination of mitochondrial membrane potential by JC-1, we adapted the method described by Cossariza and Savioli (2001) [37]. In total, 5 x 10^6^ trypomastigotes were seeded in 24-well plates in 1 mL of RPMI medium without FBS. The parasites were exposed to 0.1 or 1 μM of the different TPP^+^ derivatives for 2 hours; alternatively, the parasites were exposed to 100 μM FCCP (Sigma Aldrich, USA) for 15 min as a positive control of the uncoupling of mitochondrial respiration [38]. The cells were then washed with 500 μL of PBS and centrifuged at 1500 x g for 5 min. The parasites were then resuspended in 1 mL of PBS at 37°C. JC-1 (Molecular Probes, Applied Biosytems, USA) 1 mg/mL in DMSO was added at a final concentration of 5 μg/mL, and the mixtures were incubated for 15 min at 37°C in the dark. Next, the samples were centrifuged at 1500 x g for 5 min, and the supernatants, which contained unincorporated JC-1, were discarded. The pellet was suspended in PBS (1 mL), and fluorescence was measured using a FACSAria-III flow cytometer (BD Biosciences, USA). We used 488 nm as the excitation wavelength; the emission of JC-1 monomers was detected using the FITC filter (530/30), and the JC-1 aggregates were detected using the PE filter (585/42). Each measurement involved 5,000 events. The data were analyzed using FACSDiva 6.1 software.

### 2.8. Mitochondrial Permeability Transition Pore Opening Assessment

The opening of mitochondrial permeability transition pores was assessed using the calcein AM probe, adapting the method described by Huang *et al* (2014) [39]. Calcein-AM is a lipophilic probe that can enter the cell, including the mitochondria, and is then cleaved by cellular esterases, releasing the fluorescent probe calcein. *T*. *cruzi* trypomastigotes (Y strain, 5 x 10^6^ parasites/mL) were stained with 100 nM calcein AM (Molecular Probes, Applied Biosytems, USA) and incubated at 37°C for 15 min in the presence of 400 nM of CoCl_2_ to quench the cytosolic fluorescence of calcein. Then, the parasites were washed with PBS and resuspended in RPMI medium, without phenol red, supplemented with 0.3 mM of Ca^+2^. The parasites were then seeded in 24-well plates and exposed to the different compounds (TPP^+^-C_8_, TPP^+^-C_10_, TPP^+^-C_11_, and TPP^+^-C_12_) at 1 or 5 μM for 1 hour. As a positive control, one group was exposed to ionomycin (0.5 μM). The samples were measured using a FACS Aria III flow cytometer (BD Biosciences, USA) with the 494/517 filter to detect calcein fluorescence. For every measurement, 5,000 events were recorded. The raw flow cytometry data were analyzed using FACSDiva 6.1 software (BD Biosciences). The results were analyzed using the median fluorescence intensity (MFI) from the frequency histograms. Thus, we used the median intensity of the control group (parasites incubated with calcein AM and CoCl_2_) to establish 100% of mitochondrial fluorescence, thereby establishing percent ratios between the treatment groups and the control.

### 2.9. Statistical Analysis

For all of the experiments, the statistical significance was established at *p* < 0.05. The results represent the mean ± SD of three independent experiments, each performed in triplicate. All of the statistical analyses were carried out using GraphPad Prism (V5.0) software. Normality of the data was assessed by a D’Agostino-Pearson analysis, and a one-way analysis of variance (ANOVA) (with Dunett’s post-test) was performed to compare the experimental groups with the controls.

## Results

### 3.1. TPP^+^ Derivatives Have Cytotoxic Activity against Isolated Trypomastigotes

**Fig 2:**
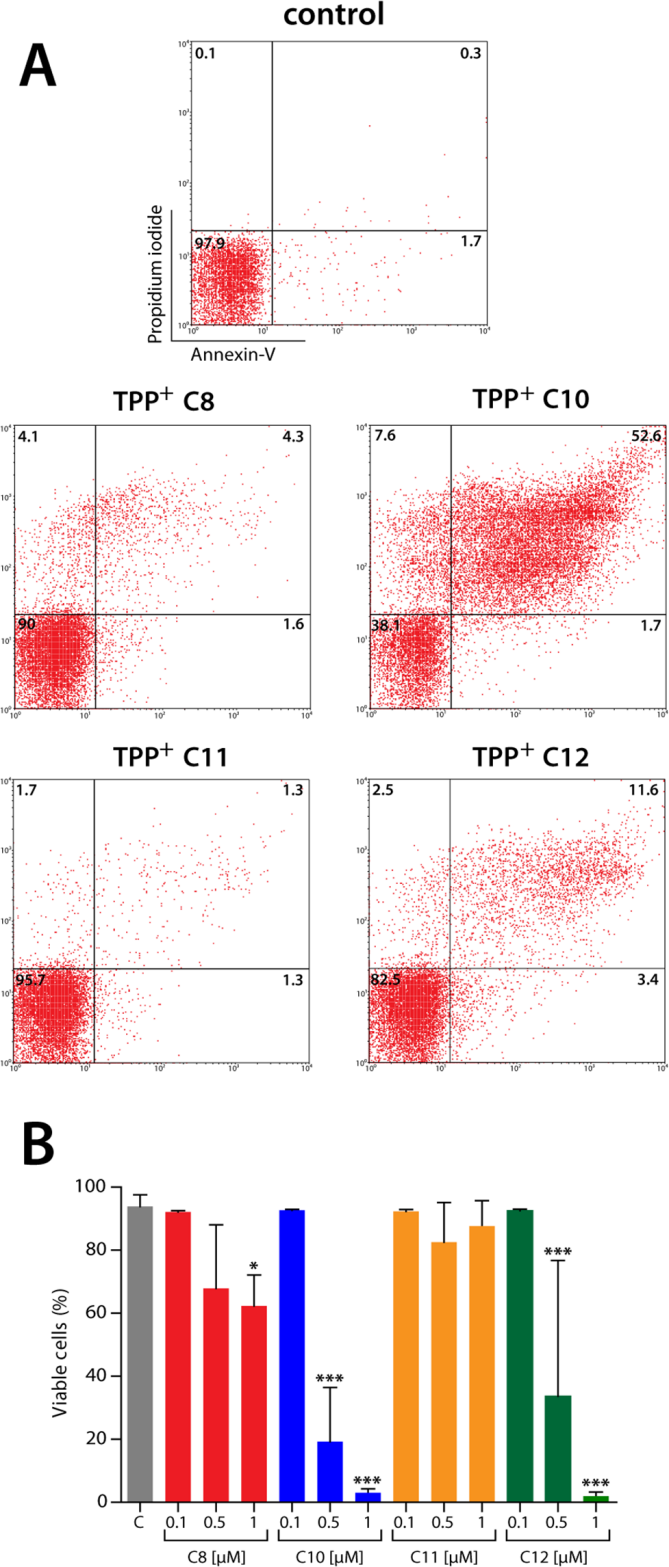
Effect of TPP^+^ derivatives on markers of cell death in *Trypanosoma cruzi*. *T*. *cruzi* trypomastigotes (Y strain, 10^7^/mL) were exposed to TPP^+^-C_8_, TPP^+^-C_10_, TPP^+^- C_11_, or TPP^+^-C_12_ at 0.1, 0.5, or 1 μM for 24 hours. The measurement of cell death markers (Annexin-V linkage and propidium iodide incorporation) was performed by flow cytometry. **A.** Representative dot plot showing the effect of TPP^+^ derivatives at 0.5 μM. The numbers within the quadrants indicate the percentage of double-negative, Annexin-V+, propidium iodide+, or double-positive cells. **B.** Quantification of viable cells (double negative) after 24 hours of exposure to the different derivatives. The bars represent the mean ± SD of three independent experiments, each performed in triplicate. *: *p* < 0.05 and ***: *p* < 0.001, compared with the control, based on one-way ANOVA with Dunnett’s post-test.

We tested four TPP^+^ derivatives (C_8_, C_10_, C_11_, and C_12_) in isolated parasites (Y strain) by MTT reduction after 24 hours of exposure to the compounds [33]. As shown in Table 1, all of the compounds had low EC_50_ values, but only the TPP^+^-C_10_ and TPP^+^-C_12_ derivatives were more potent than the reference drug nifurtimox (EC_50_: 4.1, 1.0, and 1.0 μM for nifurtimox, TPP^+^-C_10_, and TPP^+^-C_12_, respectively). Additionally, according to the same assay, the four compounds assayed were at least 10-fold more selective in parasites compared with mammalian cells (Table 1 and S1 Fig). To further analyze parasite death, we performed flow cytometry measurements of early and late apoptotic parameters after 24 hours of exposure to the TPP^+^ derivatives. As illustrated in Fig 2A, the most potent treatments were 1 μM TPP^+^-C_10_ and TPP^+^-C_12_, inducing phosphatidylserine exposure and PI uptake at 24 hours. When we quantified the viable cells (i.e., cells without annexin-V or PI positivity) at 24 hours, we found that all tested compounds decreased the number of viable cells in a concentration-dependent manner. TPP^+^-C_10_ and TPP^+^-C_12_ were the most potent compounds, inducing a significant decrease in the number of viable cells at concentrations of 0.5 and 1 μM (Fig 2B).

**Table 1:** Effect of triphenylphosphonium (TPP^+^) derivatives on the viability of *T*. *cruzi* trypomastigotes and VERO cells. *T*. *cruzi* (Y strain) trypomastigotes and VERO cells were exposed to TPP^+^-C_8_, TPP^+^-C_10_, TPP^+^-C_11_, or TPP^+^-C_12_ for 24 hours, and viability was measured using the MTT assay. The data are presented as the means ± SD of three independent experiments. The curves from which the values were obtained are shown in the S1 Fig ^a^: *p* < 0.001 compared with nifurtimox. The *p* value was calculated using one-way ANOVA and Dunnett’s post-test.

Compound	EC_50_ (μM)		Selectivity index (VERO/*T*. *cruzi*)
	*T*. *cruzi* trypomastigote	VERO cells	
Nifurtimox	4.1 ± 0.9	> 100	> 24
TPP^+^-C_8_	4.8 ± 0.9	> 100	> 21
TPP^+^-C_10_	1.0 ± 0.6^a^	11.3 ± 0.1	11.3
TPP^+^-C_11_	9.2 ± 0.1^a^	> 100	> 11
TPP^+^-C_12_	1.0 ± 0.7^a^	10.9 ± 2.4	10.9

### 3.2. TPP^+^ Derivatives Reduce the Parasite Load in Infected Cells

**Fig 3:**
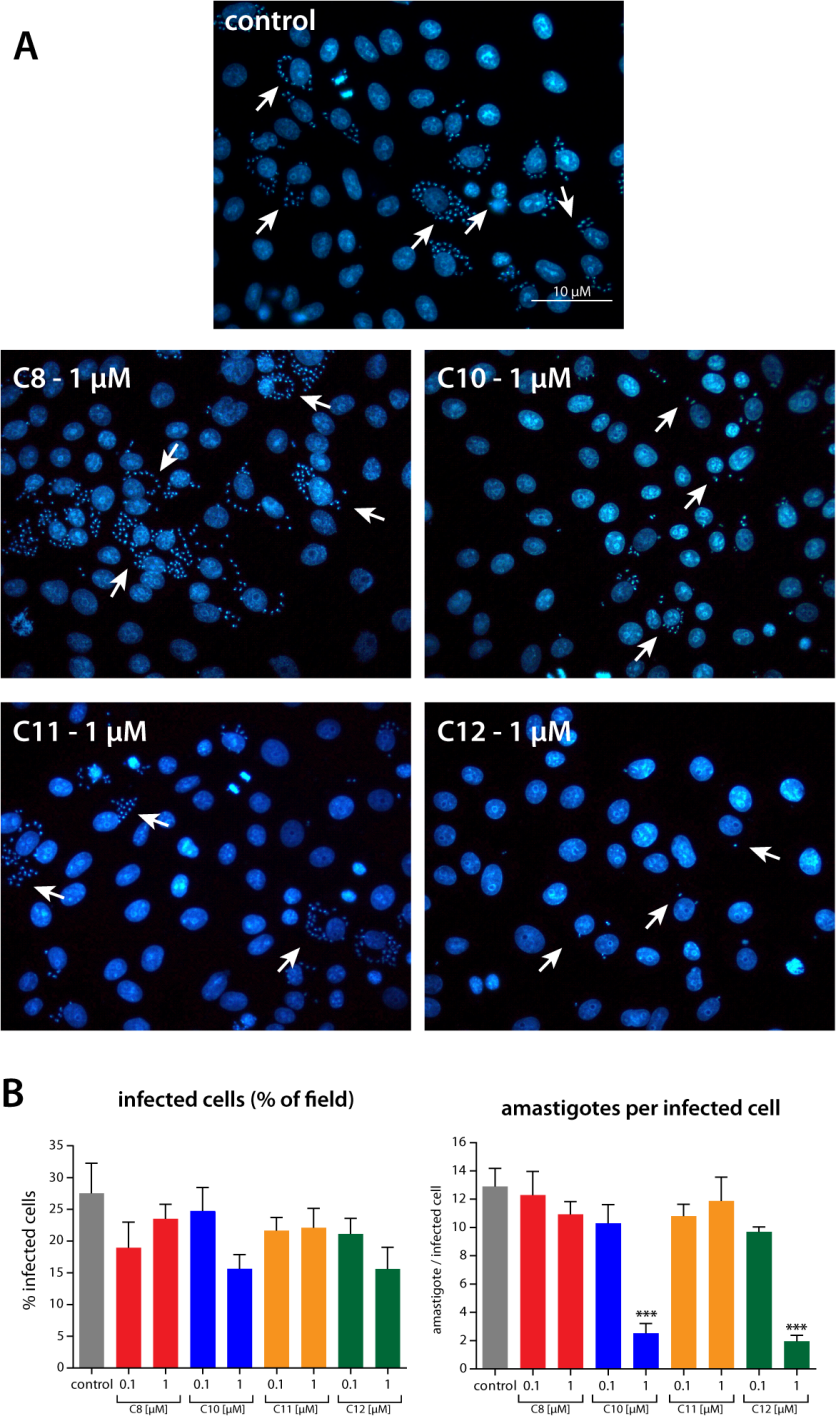
Effect of TPP^+^ derivatives on the amastigote number in *T*. *cruzi*-infected cells. VERO cells were infected with *T*. *cruzi* trypomastigotes (Y strain). The cells were treated for 48 hours with the different derivatives, and the parasite load was then assessed by DAPI staining and fluorescence microscopy visualization. **A.** Representative images showing the effect of the different TPP+ derivatives on the intracellular amastigote number. All of the compounds shown were used at 1 μM. The white arrows show amastigote nuclei stained with DAPI. **B.** Quantification of the effect of TPP^+^ derivatives on VERO cells infected with *T*. *cruzi*. For each condition, performed in triplicate, at least five images were taken. The left panel shows the percentage of infected cells in each microscopic field photographed. The right panel shows the number of amastigotes per infected cell in each microscopic field photographed. For all of the graphs in the figure, the results are expressed as the mean ± SD of three independent experiments, each performed in triplicate. ***: *p* < 0.001 compared with the control, based on one-way ANOVA with Dunnett’s post-test.

**Fig 4:**
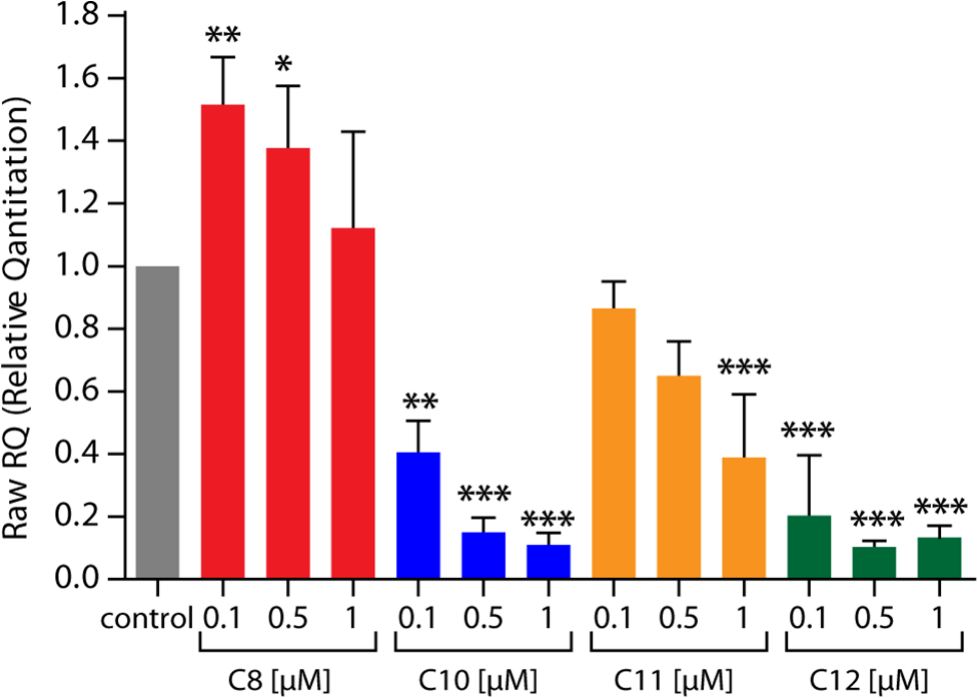
Effect of TPP^+^ derivatives on the parasite load in *T*. *cruzi*-infected cells. RAW 264.7 cells were infected with *T*. *cruzi* trypomastigotes (Y strain); the cells were treated for 48 hours with the different derivatives, and the parasite load was assessed by qPCR. The results are expressed as the relative quantification of the parasite DNA and mammalian DNA, with a ratio of 1 assigned to the control. Treated cells were compared with the control using the ^∆∆^C_T_ method. *: *p* < 0.05; **: *p* < 0.01; and ***: *p* < 0.001, compared with the control, based on one-way ANOVA with Dunnett’s post-test.

To assess the effect of the derivatives on intracellular amastigotes, we evaluated their effects in two models of *in vitro* infection. First, we quantified amastigotes directly by microscopic examination of infected VERO cells treated with the different derivatives at 0.1 and 1 μM (Fig 3). The TPP^+^-C_10_ and TPP^+^-C_12_ derivatives were the most potent compounds, yielding a significant reduction in parasite load in these cells (Fig 3A). When we quantified the effect of the compounds, we found that no single treatment reduced the number of infected cells; however, 1 μM TPP^+^-C_10_ and TPP^+^-C_12_ significantly reduced the number of amastigotes per infected cell, namely, from 12.9 ± 2.2 to 2.5 ± 1.1 for TPP^+^-C_10_ and 2.0 ± 0.7 for TPP^+^-C_12_ (Fig 3B). In addition, we evaluated the parasite load in infected RAW cells (murine macrophages) by qPCR at 48 hours after treatment with the TPP^+^ derivatives (Fig 4). Unexpectedly, the lowest concentrations of TPP^+^-C_8_ increased the parasite load, whereas the TPP^+^-C_10_, TPP^+^-C_11_, and TPP^+^-C_12_ derivatives reduced the parasite load in a concentration-dependent manner. Again, TPP^+^-C_10_ and TPP^+^-C_12_ showed the most potent effect, significantly reducing the parasite load at all of the assayed concentrations.

### 3.3. TPP^+^ Derivatives Induce Mitochondrial Changes in *T*. *cruzi*

**Fig 5:**
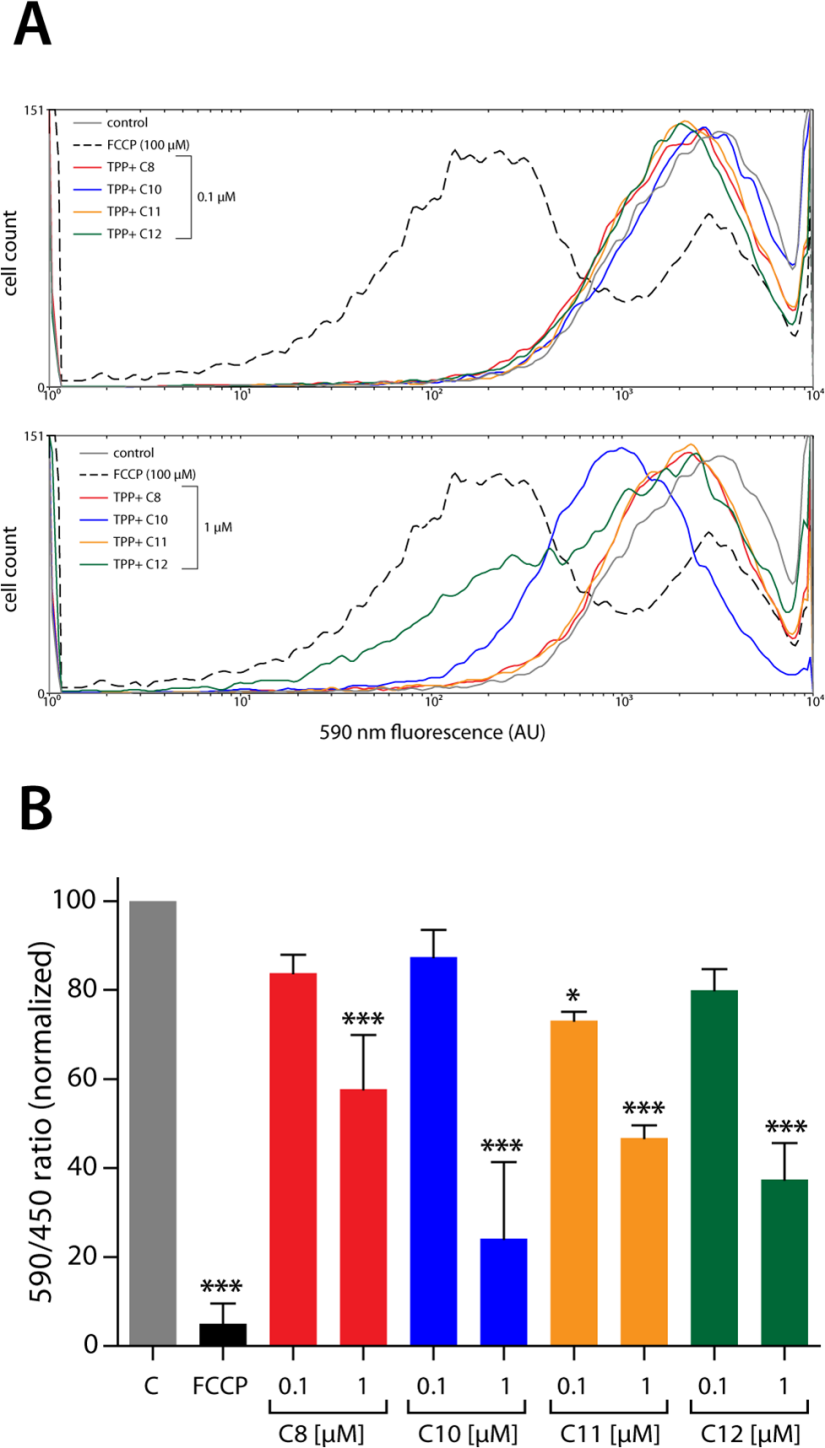
Effect of TPP^+^ derivatives on the mitochondrial transmembrane potential (ΔΨ_m_) of *T*. *cruzi*. *T*. *cruzi* trypomastigotes were exposed to the different TPP^+^ derivatives for 2 hours, and ΔΨ_m_ was evaluated through JC-1 fluorescence using a flow cytometer. **A.** Representative frequency histogram of the fluorescence emitted at 590 nm. Upper panel: trypomastigotes exposed to 0.1 μM of the TPP^+^ derivatives. Lower panel: trypomastigotes exposed to 1 μM of the TPP^+^ derivatives. In both panels, FCCP (100 μM, 15 minutes before measurement) is shown as a positive control. Control: grey line, TPP^+^-C_8_: red line, TPP^+^-C_10_: blue line, TPP^+^-C_11_: orange line, TPP^+^-C_12_: green line, FCCP: black dashed line. AU: Arbitrary Units of Fluorescence. **B.** Quantification of the ratio between the 590 and 490 nm fluorescence emissions in trypomastigotes exposed to the TPP^+^ derivatives. The ratios were calculated using the fluorescence medians obtained from the histograms. The results are expressed as the mean ± SD of three independent experiments, each performed in triplicate. *: *p* < 0.05 and ***: *p* < 0.001, compared with the control, based on one-way ANOVA with Dunnett’s post-test.

To assess the mitochondrial damage induced by the TPP^+^ derivatives, we measured changes in the JC-1 aggregation state after 2 hours of exposure to the compounds. JC-1 is a fluorescent probe that aggregates in the mitochondrial matrix in healthy mitochondria, generating red fluorescence; the non-aggregated form of JC-1 remains in the cytosol, producing a green fluorescence. When the mitochondrial transmembrane potential (ΔΨ_m_) decreases, the red fluorescence is lost, leading to a change in the red/green ratio that is associated with a change in mitochondrial health. Fig 5A shows representative frequency histograms of the red aggregates in parasites. The upper panel of Fig 5A shows that none of the compounds at 0.1 μM induced changes in red fluorescence; however, red fluorescence was nonetheless observed for the positive control, FCCP. The bottom panel of Fig 5A shows that TPP^+^-C_10_ and TPP^+^-C_12_ at 1 μM induced mitochondrial changes, though to a lower extent than FCCP. When we quantified the fluorescence ratio induced by the compounds (Fig 5B), we found that all compounds at 1 μM significantly decreased the red/green ratio, indicating an alteration in ΔΨ_m_. Once again, TPP^+^-C_10_ and TPP^+^-C_12_ showed more potent effects than the TPP^+^-C_8_ or TPP^+^-C_11_ derivative.

**Fig 6:**
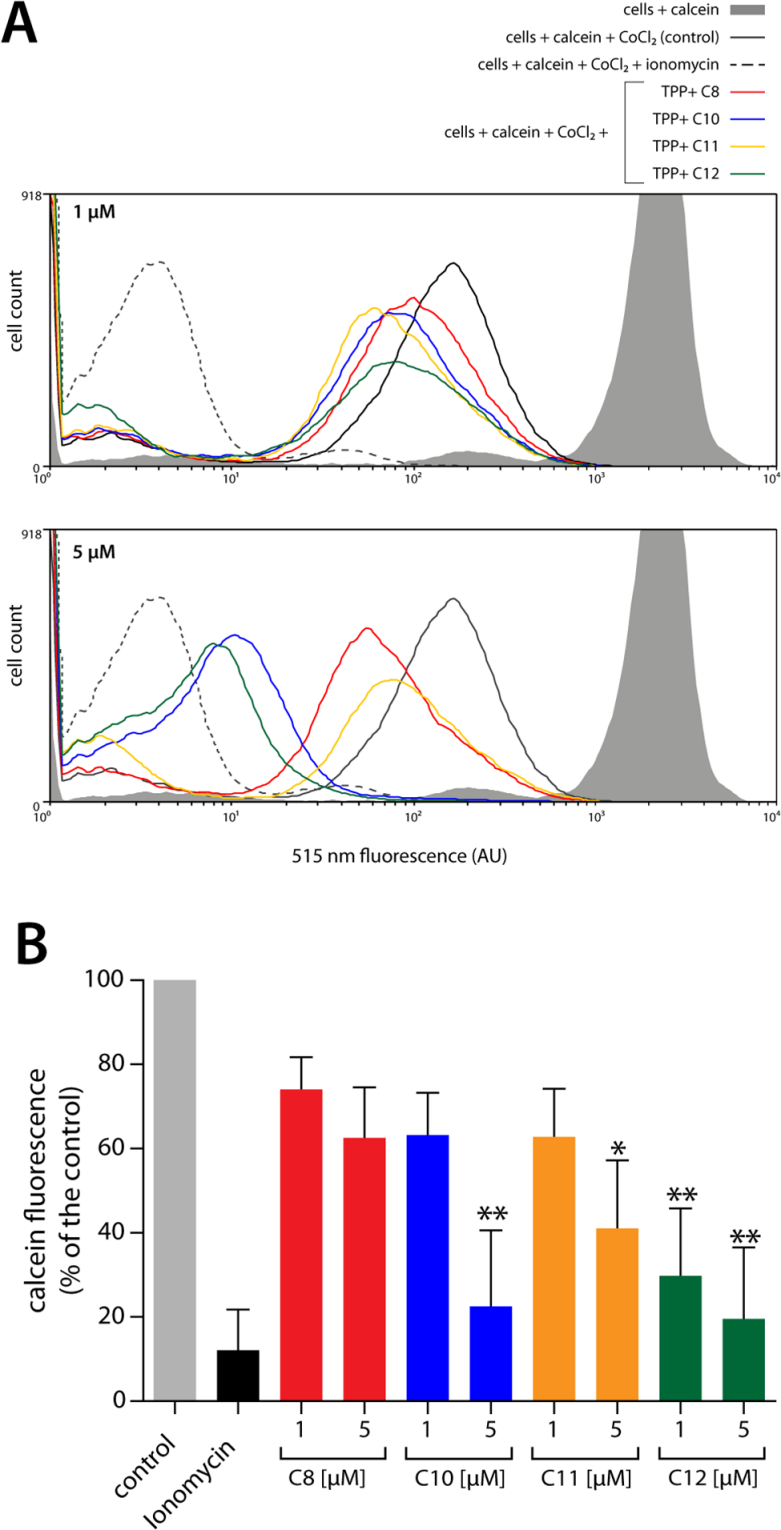
Effect of TPP^+^ derivatives on the opening of mitochondrial permeability transition pore. *T*. *cruzi* trypomastigotes were loaded with calcein AM dye and CoCl_2_ and exposed to the different TPP^+^ derivatives for 1 hour. Transition pore opening was then evaluated using a flow cytometer based on the quenching of mitochondrial calcein fluorescence. **A.** Representative frequency histogram of the fluorescence emitted at 515 nm. Both panels show calcein fluorescence in intact cells (grey area). Cytosolic fluorescence was quenched by the addition of CoCl_2_, allowing visualization of the fluorescence from *T*. *cruzi* mitochondria (black line). The median fluorescence intensity of cells incubated with calcein AM and CoCl_2_ was used as a control. As a positive control, we incubated the cells with 0.5 μM ionomycin (black dashed line). Parasites were also incubated with TPP^+^-C_8_ (red line), TPP^+^-C_10_ (blue line), TPP^+^-C_11_ (orange line), and TPP^+^-C_12_ (green line). Upper panel: trypomastigotes exposed to 1 μM of the TPP^+^ derivatives. Lower panel: trypomastigotes exposed to 5 μM of the TPP+ derivatives. AU: Arbitrary Units of Fluorescence. **B.** Quantification of the median fluorescence at 515 nm. The results were calculated using the median fluorescence obtained from the histograms and normalized assuming 100% fluorescence of the controls. The results are expressed as the mean ± SD of three independent experiments, each performed in triplicate. *: *p* < 0.05 and ***: *p* < 0.001, compared with the control, based on one-way ANOVA with Dunnett’s post-test.

To further relate the observed mitochondrial damage with apoptotic phenomena, we studied transition pore opening in isolated parasites stained with the calcein AM probe and exposed to the TPP^+^ derivatives for 1 hour. In this measurement, fluorescence intensity is related to the presence of calcein in mitochondria; thus, reduced fluorescence indicates that calcein has leaked from the mitochondria due to permeability transition pore opening. Fig 6A presents representative frequency histograms of the effects of the TPP^+^ derivatives at 1 μM (upper panel) or 5 μM (lower panel). After 1 hour of incubation with 1 μM of the compounds, no changes were observed in mitochondrial calcein. However, 5 μM of the compounds induced calcein leakage, indicating the opening of mitochondrial pores. Among the four derivatives, TPP^+^-C_10_ and TPP^+^-C_12_ were the most potent (Fig 6A). Upon quantifying the effects of the compounds (Fig 6B), we found that only C_12_ induced a significant decrease in fluorescence at 1 μM (29% of the control, *p*<0.01). At 5 μM, TPP^+^-C_10_, TPP^+^-C_11_, and TPP^+^-C_12_ induced significant changes, with TPP^+^-C_10_ and TPP^+^-C_12_ being the most potent compounds (TPP^+^-C_10_: 22%, TPP^+^-C_11_: 41%, and TPP^+^-C_12_: 20% of the control).

## Discussion

The electron transport chain of *T*. *cruzi* has features that make it an interesting drug target. One of these features is the presence of an alternative complex I that is insensitive to rotenone. The activity of complexes II, III, and IV was demonstrated in 1970. Interestingly, KCN, a classic inhibitor of complex IV, does not completely inhibit *T*. *cruzi* respiration, indicating the existence of an alternative terminal oxidase. Thus, given that *T*. *cruzi* has only one mitochondrion, alternative oxidases and a rudimentary antioxidant defense, this organelle is a potential candidate for pharmacological intervention in this organism [40–42].

There are several compounds with known anti-*T*. *cruzi* activity and with mechanisms of actions related to mitochondrial toxicity. Naphthoquinones and their derivatives lead to alterations in the mitochondrial membrane potential, induce oxidative stress, and block the activity of complex III [43]. Helenalin and dehydroleucodine, which are natural sesquiterpene lactones, induce cellular death in *T*. *cruzi* through mitochondria-induced apoptosis [44]. Propolis derivatives and phenolic compounds show mitochondrial effects against *T*. *cruzi* trypomastigotes, especially with regard to the kinetoplast [45]. Geranylgeraniol, an alcoholic terpene, causes mitochondrial swelling in *T*. *cruzi* trypomastigotes and amastigotes, and induces a significant decrease in the mitochondrial transmembrane potential [45]. However, most of the available data have been obtained using the epimastigote form of the parasite, which frequently requires higher drug concentrations than trypomastigotes. Here, we show that our compounds are almost equal in potency to nifurtimox (for TPP^+^-C_8_ and TPP^+^-C_11_), with the most potent compounds being four times more potent than the reference drug. In addition, it is important to note that all of the mitochondrial effects were observed under conditions similar to those used in our viability experiments.

GA has previously been assayed as a trypanocidal agent against *Trypanosoma brucei* [46, 47]; in these models, the EC_50_ for GA is always over 30 μM (at least 30-fold higher than that used for our more effective compounds). In addition, GA has been assayed as a potential “blood disinfectant” for blood contaminated with *Trypanosoma cruzi* [27]. In that model, GA exerts its effects in the millimolar range. However, the addition of decyl- or lauryl- hydrocarbon carbonate chains was reported to lead to an uncoupling of the oxidative phosphorylation system due to decreases in ΔΨ_m_, and concentrations one order of magnitude less were required in mammalian cells [27, 31]. Among all of the known GA derivatives, one of the most assayed compounds is (-)-epigallocatechin 3-O-gallate (EGCg), which has been demonstrated to have *in vitro* efficacy against *Leishmania brasiliensis*. However, the effect on viability is reached at concentrations over 60 μM, a concentration at which EGCg also induces mitochondrial alteration and ROS production [48]. EGCg has also been assayed against *T*. *cruzi* epimastigotes. In these experiments, the antiproliferative effect of EGCg was only observed at concentrations over 500 μM [49]. The concentration range over which our compounds are active indicates that these are the most active GA derivatives obtained to date. There are data regarding the improved activity of GA esters that are more lipophilic (due to a longer alkyl chain); however, esters with alkyl chains that have more than 14 carbon atoms begin to behave as detergents in the cell membrane due to the hydrocarbon chain size (i.e., cut-off effect). This effect makes such compounds extremely toxic and less active [50]. In our series, improved activity was observed with respect to chain length and, thus, the lipophilicity of the compounds. However, it is necessary to better understand the results obtained for TPP^+^-C_11_, which showed less activity than TPP^+^-C_10_ despite having a longer chain.

It should be noted that the range of concentrations at which these derivatives exert their cytotoxic action is similar to recently described lipophilic cations [51–53]. Furthermore, at the concentrations tested, our derivatives are different from other lipophilic cations because the pharmacophore group in our case exerts an uncoupling effect, unlike what occurs with previously described compounds, in which the pharmacophore groups have the ability to bind to any component of the electron transport chain and inhibit some complexes. This effect prevails over the effect of the load of TPP^+^ group, which was shown in some cases to exert an uncoupling effect itself because the group counteracts the negative charge at the level of the mitochondrial matrix (uncoupling effect), resulting in a decrease in potential. Furthermore, there is evidence that molecules that perturb the electron transport chain induce a rise in mitochondrial ROS production in *T*. *cruzi*, which makes these compound less selective and safe [54, 55].

Finally, the TPP^+^-C_10_ derivative has been demonstrated to be safe at 15 mg/kg in mouse models [31]; thus, the TPP^+^-C_10_ and TPP^+^-C_12_ derivatives are good candidates for further animal tests with regard to their efficacy against Chagas disease.

## Supporting Information

S1 Fig Viability curves for *T*. *cruzi* trypomastigotes exposed to TPP^+^ derivatives. *T*. *cruzi* trypomastigotes (Y strain) were cultured in RPMI medium at a density of 10^7^ parasites/mL and exposed to the different TPP+ derivatives or nifurtimox (as positive control) for 24 hours. Viability was measured by MTT reduction. Data are shown by experiment, and each experiment was performed in triplicate. For each experiment, the IC_50_ value was obtained fitting the data to the dose-response (four parameters) equation, using the GraphPad Prism Software (V 5.0). The IC_50_ value of each drug was finally obtained calculating the mean and standard deviation (SD) between the IC_50_ obtained for each compound.
(TIF)
