# Supplementary figures and images for: Novel Gallate Triphenylphosphonium Derivatives with Potent Antichagasic Activity
Source: PLoS One. 2015 Aug 28;10(8):e0136852. doi: 10.1371/journal.pone.0136852 (PMC4552745; doi:10.1371/journal.pone.0136852)

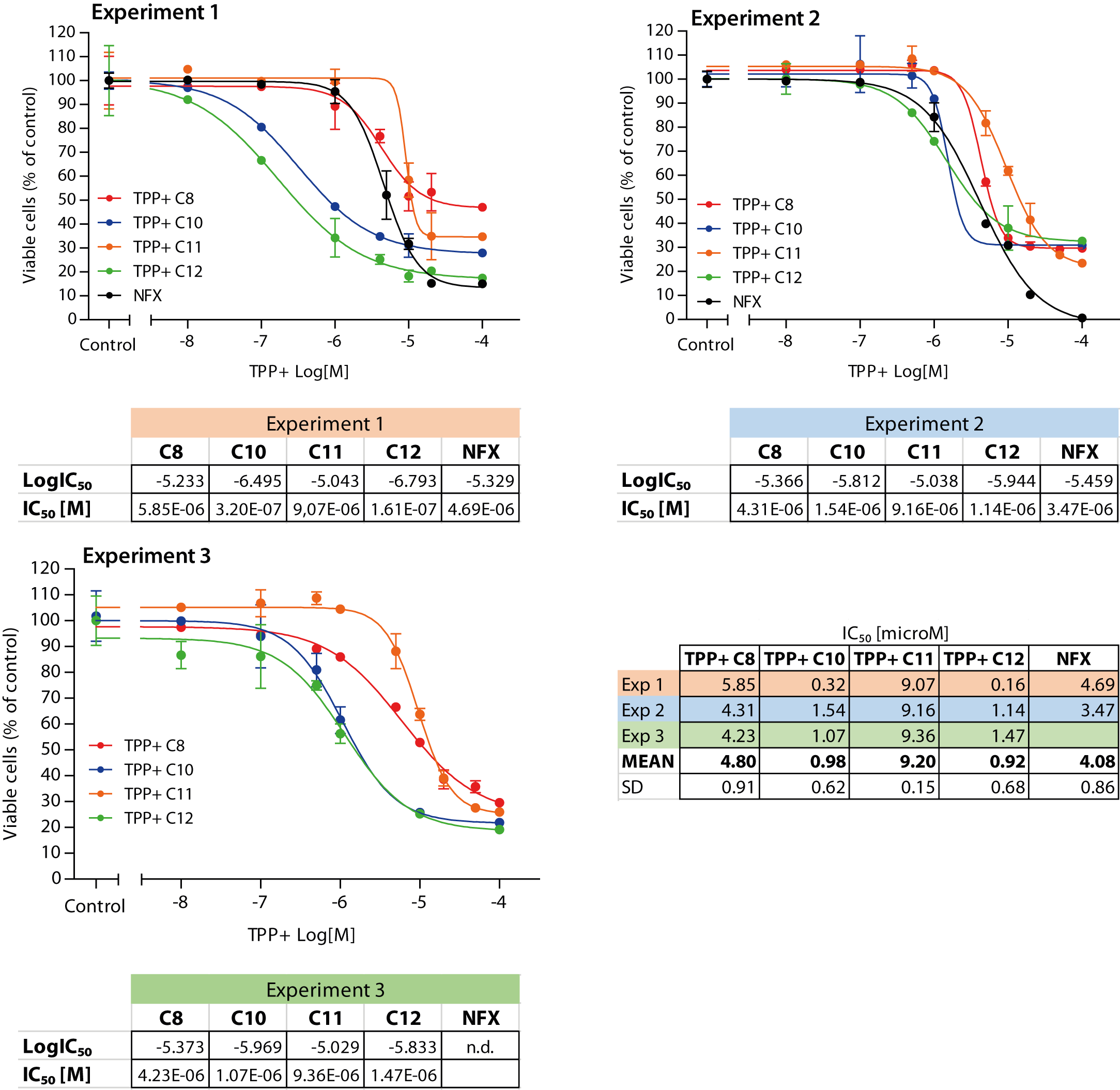

Supplement: S1 Fig — T. cruzi trypomastigotes (Y strain) were cultured in RPMI medium at a density of 107 parasites/mL and exposed to the different TPP+ derivatives or nifurtimox (as positive control) for 24 hours. Viability was measured by MTT reduction. Data are shown by experiment, and each experiment was performed in triplicate. For each experiment, the IC50 value was obtained fitting the data to the dose-response (four parameters) equation, using the GraphPad Prism Software (V 5.0). The IC50 value of each drug was finally obtained calculating the mean and standard deviation (SD) between the IC50 obtained for each compound. (TIF) [file pone.0136852.s001.tif]
